# 3C-SiC Growth on Inverted Silicon Pyramids Patterned Substrate

**DOI:** 10.3390/ma12203407

**Published:** 2019-10-18

**Authors:** Massimo Zimbone, Marcin Zielinski, Corrado Bongiorno, Cristiano Calabretta, Ruggero Anzalone, Silvia Scalese, Giuseppe Fisicaro, Antonino La Magna, Fulvio Mancarella, Francesco La Via

**Affiliations:** 1 CNR-IMM, V. S.Sofia 64, 95129 Catania, Italy; massimo.zimbone@imm.cnr.it; 2 Savoie Technolac-Arche Bat.4, Allée du Lac d’Aiguebelette, BP 267, 73375 Le Bourget du Lac CEDEX, France; mzielinski@novasic.com; 3 CNR-IMM, Zona Industriale VIII Strada 5, 95121 Catania, Italy; Corrado.Bongiorno@imm.cnr.it (C.B.); Silvia.Scalese@imm.cnr.it (S.S.); giuseppe.fisicaro@imm.cnr.it (G.F.); antonino.lamagna@imm.cnr.it (A.L.M.); francesco.lavia@imm.cnr.it (F.L.V.); 4 STMicroelectronics, Stradale Primosole, 50, 95121 Catania, Italy; ruggero.anzalone@st.com; 5 CNR-IMM, Via Gobetti 101, I-40129 Bologna, Italy; mancarella@bo.imm.cnr.it

**Keywords:** 3C-SiC, ISP, compliant substrate, stacking faults, anti-phase boundary, heteroepitaxy, growth rate, STEM, micro Raman, SEM

## Abstract

This work reports on the properties of cubic silicon carbide (3C-SiC) grown epitaxially on a patterned silicon substrate composed of squared inverted silicon pyramids (ISP). This compliant substrate prevents stacking faults, usually found at the SiC/Si interface, from reaching the surface. We investigated the effect of the size of the inverted pyramid on the epilayer quality. We noted that anti-phase boundaries (APBs) develop between adjacent faces of the pyramid and that the SiC/Si interfaces have the same polarity on both pyramid faces. The structure of the heterointerface was investigated. Moreover, due to the emergence of APB at the vertex of the pyramid, voids buried on the epilayer form. We demonstrated that careful control of the growth parameters allows modification of the height of the void and the density of APBs, improving SiC epitaxy quality.

## 1. Introduction

In the last few years, significant interest in silicon carbide (SiC) has emerged due to the possibility of high power and high current device fabrication. The interest is driven by the automotive industry. Indeed, transition from gasoline to hybrid and to fully electric vehicles is occurring. This transition is part of a green revolution, which is going to be the trigger for the development of new devices not only for electric motors but also for high voltage energy saving applications driving the society to a more sustainable economy. Despite the enormous interest and despite the enormous high power device market growth, the material is scarcely mature for the standards required in the microelectronic industry and is extremely expensive. The main limitation for device manufacture comes from the wafer fabrication that allows the presence of a high density of defects in the epilayer.

Actually, the most controllable and less defective polytype is hexagonal 4H-SiC silicon-carbide but, it has two main disadvantages with respect to the cubic polytype (3C-SiC): (1) it cannot be grown on a silicon substrate, making it extremely expensive, and (2) it has lower channel mobility in Metal Oxide Semiconductor Field Effect Transistor (MOSFET) than 3C-SiC [1,2]. The world of power electronics and sensors for high temperatures and harsh environments is opening up to 3C-SiC [3,4,5]. The use of 3C-SiC, instead of 4H-SiC, would reduce the costs, and it would boost the ongoing transition to a new and more sustainable economy. For these reasons, the growth and fabrication of a high-quality 3C-SiC epilayer and bulk wafers are becoming very important. The most crucial issues that hinder the use of 3C-SiC are the high number of defects in both bulk and epilayer (such as stacking faults and grain boundaries) and the high bow of the Si wafer after the growth of the SiC. These issues arise from the different lattice parameters and the difference in the thermal expansion coefficient [6,7]. The latter creates a network of dislocations at the interface that evolves into stacking faults (SFs) and grain boundaries (GBs) in the bulk, and the former causes the build-up of internal stress that can be released by the formation of several classes of extended defects, including also SFs and GBs [8,9,10,11,12,13]. 

So far, several different approaches have been proposed to eliminate alleviate the above-cited problems. One of the most interesting techniques is the use of compliant substrates. The compliant substrate should be able to better adapt the SiC interface with the Si one and thus reduce the defectivity in the SiC layer grown on Si. Among these approaches, we cite the use of the SiGe buffer layer [14] or the growth on a patterned substrate as inverted silicon pyramids (ISP) [15] silicon pillars [16] or the use of porous silicon (p-Si) as substrate [17]. 

The most important improvement in the 3C-SiC on Si quality is due to Nagasawa et al. in early 2000 [18]. They proposed to cover the (001) Si surface with an undulant structure aligned in the [110] direction. This structure is able to eliminate anti-phase boundaries and twins boundaries. Moreover, the same team used the switch-back epitaxy methods to grow a 200 μm thick 6-inch wafer with a remarkable quality [19], and further, they demonstrated a good performance of vertical and lateral MOSFET [20,21]. Nevertheless, despite the obtained improvements, the density of SFs remains quite high and cannot be reduced below 120 cm^−1^ [22,23]. 

In the present paper, we investigate a particular compliant substrate able to reduce the number of SFs in the epilayer surface. The SFs lie on {111} planes and can interact with each other in several ways. Some of these interactions stop the propagation of SFs in the epilayer and thus reduce the number of defects approaching the surface. We propose a compliant substrate able to promote the self-annihilation of the SFs’ mechanism. In particular, we patterned the Si surface with an array of inverted silicon pyramids (ISP) having their squared bases along the [110] direction covering the whole 6-inch (001) Si wafer. We investigated the morphology of the surface and recognized the presence of extended defects, such as anti-phase boundaries (APB), related to the patterning structure. We further observed the formation of voids buried on the epilayer that form due to the merging of APB in the vertex of the pyramid. We studied how to control the height of the void and the density of APB by exploiting an APB elimination mechanism.

## 2. Materials and Methods

ISP were manufactured on 500 μm thick (001) Si wafer by Deep UV contact lithography. The structures were 700 nm wide square geometries with a 1.4 µm pitch. A thin layer of stoichiometric silicon nitride deposited by LPCVD with a thin layer of thermal Silicon dioxide as a buffer layer was used as a hard mask. The layer was etched by a fluorine-based plasma. The silicon substrate was anisotropically etched by a 45 wt.% KOH @ 70 °C solution. 

To bring ISP structures as close as possible, the etching time was extended by exploiting the non-zero etching rate of the KOH on the <111> plane. The gap among the structures after the wet etching was between 60 and 100 nm on the whole substrate. 

The growth process was performed at the NOVASIC (Nice France) CVD reactor, using silane (SiH_4_), propane (C_3_H_8_), and H_2_ as silicon, carbon precursors, and gas carrier, respectively. The carbonization step (or buffer layer) was performed at 1120 °C and with a C_2_H_4_ flow rate of 120 sccm while flowing the H_2_ gas carrier at 100 slm [24]. Deposition temperature of 1380 °C, a C/Si ratio (the ratio between silicon and carbon atoms) of 1.6, and Si/H_2_ ratio between 0.032% were used. The cubic polytype was the only possible structure obtained at low growth temperature on silicon. The reactor chamber pressure was held at 100 mbar during the entire growth process, if not specified. The growth rates used were maintained to 1 μm/h, if not specified. In a specific set of experiments, N_2_ gas was introduced in the reaction chamber every 10 min during the growth. For a better evaluation of the impact of the ISP substrate on the resulting 3C-SiC films, each growth process was conducted simultaneously on both ISP and conventional (001) Si. 

The film thickness was measured by Fourier transform infrared spectroscopy. Scanning electron microscopies SEM are performed with ZEISS SUPRA 35, (Carl Zeiss, Oberkochen, Germany). Scanning transmission electron microscopes (STEM) images are acquired on a ARM200F probe Cs-corrected TEM (Jeol, Tokyo, Japan), equipped with a cold field emission gun (FEG) and working at 200 kV with a nominal point resolution of 0.68 Å. Under experimental conditions, the intensities observed in the STEM micrographs were roughly proportional to the atomic number Z and enabled a direct interpretation.

## 3. Results and Discussion

### 3.1. Formation of GB on ISP

**Figure 1:**
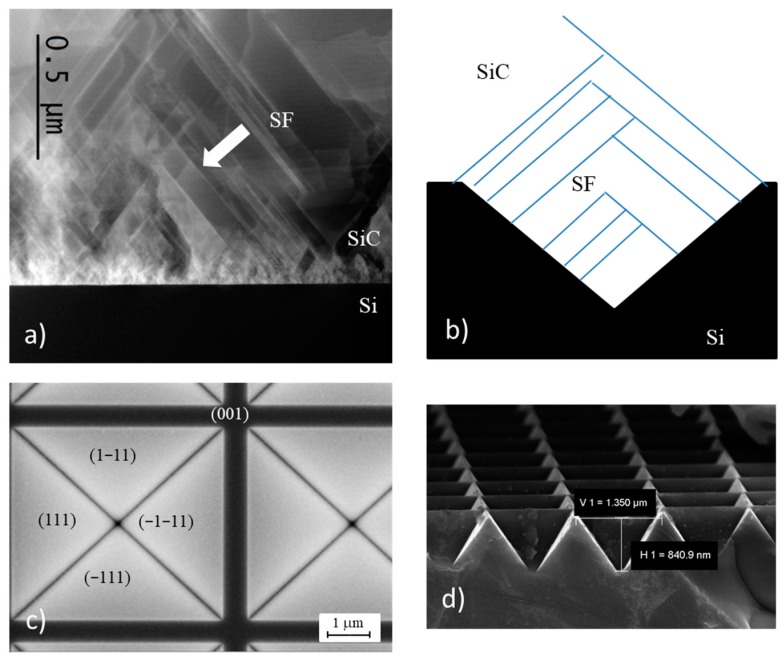
(**a**) Scanning TEM cross-section image of cubic silicon carbide (3C-SiC) grown on a flat silicon wafer. Bright lines indicated by the arrow are stacking faults (SFs); (**b**) pictorial cross-section view of SF self-annihilation mechanism. The black region represents silicon while white SiC. Blue lines are SFs; (**c**) SEM plan view of the 5 × 5 µm^2^ pyramid. The four (111) planes of the pyramid are indicated together with the (001) zone between two pyramids; (**d**) Cross view SEM image of 1 × 1 µm^2^ pyramid.

In Figure 1a, a Scanning TEM cross-section image of 3C-SiC grown on a flat silicon wafer is shown. A huge number of defects are evident near the interface, but moving away from the interface, the defectivity decreases. At about 150 to 200 nm from the interface, there are evident bright lines. The ones laying in the <112> directions are individuated, in literature, to be stacking faults while curved lines are grain boundaries [19,20,21,22,23,25]. SFs can interact with each other. They can (1) cross each other creating an X shaped defect called Forest dislocation, (2) self-annihilate creating a structure called Lomer dislocation, or (3) terminate on a pre-existing SF creating a “λ shaped defect” [11,12]. In the case of “λ shaped defect,” or in the case of Lomer dislocation, the interaction of SF lead to a decrease in SFs number that finally reaches the surface.

The annihilation of SFs can be promoted by using ISPs as a substrate. In Figure 1b, we propose a pictorial cross-section view of this compliant substrate. Black and white regions indicate silicon and silicon carbide, respectively. Blue lines are SFs. SFs lying, for example, in the (111) plane cross the SF coming from the opposite sides of the pyramid (i.e., lying in the (−1−11) plane) and self-annihilate, reducing the defectivity of the material. 

The SFs reduction rate follows a self-annihilation mechanism inversely proportional to SF density. The use of an ISP compliant substrate makes it possible to obtain an important reduction in SF concentration just in thin epilayer growth through its characteristic geometry, which is able to concentrate SFs in small regions, thus increasing the probability of SF annihilation as the mutual closure mechanism is stimulated. Therefore, the ISP substrate turns out to be particularly advantageous. Taking into account that the concentration of SFs reaches a saturation value (due to a trade-off mechanism in the generation/annihilation process) an ISP compliant substrate allows SFs to reach saturation density in shorter epilayer thicknesses with respect to (100), (111) flat substrates, or undulant silicon [15,26].

Following this idea, we investigated the effect of ISP size and pitch on the 3C-SiC epilayer properties. In Table 1, the main structural features (size and pitch) of the manufactured pyramids are tabulated, and in Figure 1c,d, plan and cross-view of the ISP are shown respectively. In Figure 1c, we show, as an example, the plan view of a 5 × 5 µm^2^ pyramid. The four (111) faces of the pyramid are highlighted. In the same figure, the (001) zone, between two pyramids, is clearly observed. In Figure 1d, a cross view of a 1 × 1 µm^2^ sized pyramid is shown. The thin layer hard mask has been removed by a 15% HF solution. After the long over etching by KOH, the gap among the ISP structures is lower than 100 nm. 

**Figure 2:**
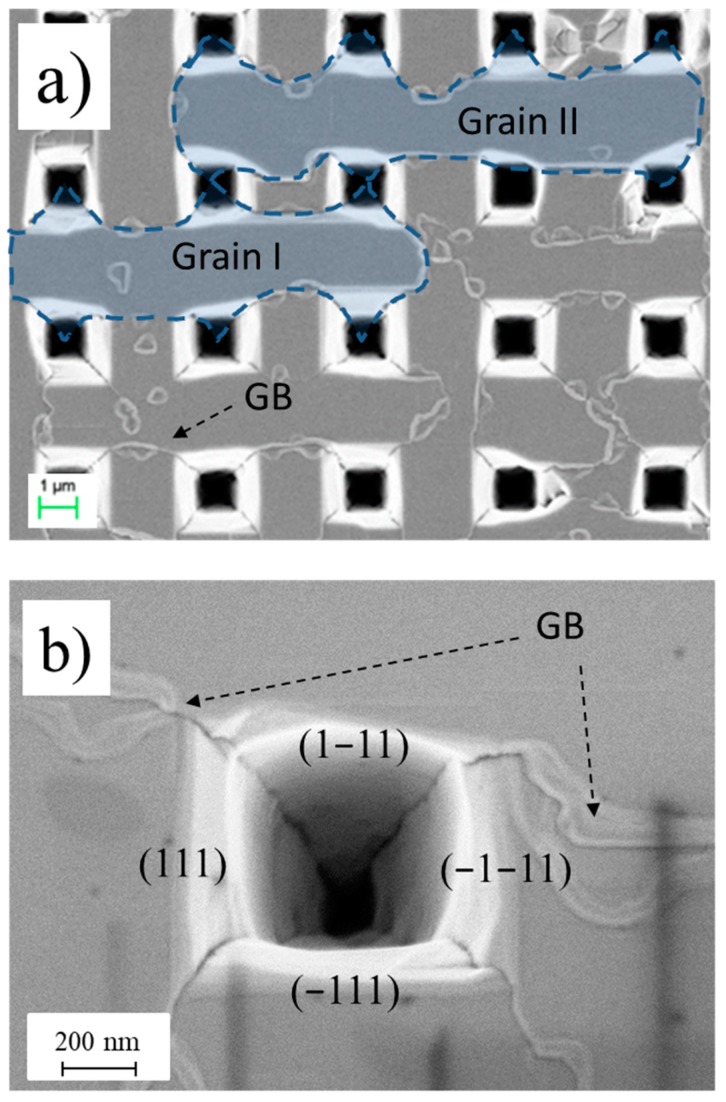
(**a**) Plan view SEM images of 2 μm 3C-SiC film grown on 3 × 3 μm^2^ inverted silicon pyramids (ISP). As an example, the contour of two grains is highlighted in blue; (**b**) High magnification image of the pyramid showing GBs and pyramid faces.

Figure 2a shows a plan view SEM image of the 3C-SiC film grown on ISP. The thickness of the SiC layer was 2 μm, and the pyramid size was 5 × 5 μm^2^. In this experimental condition, the silicon carbide layer was thin and did not fill the pyramid; thus, a structure with holes formed. In this image, the presence of small black wavy lines between pyramids can be noted. These lines create small domains or grains between pyramids. As an example, two grains are highlighted in light blue in the same figure. The contour of the grain is a grain boundary (GB). 

It is worth noting that the GBs (dotted blue lines) are always connected to the edges and the vertex of the pyramid. A very interesting feature that is depicted in Figure 2a is that the grains contain the (1−11) and (−111) planes (up and down pyramid faces) or (111) and (−1−11) (left and right faces). We are not able to report a domain that contains two adjacent sides of the pyramid as, for example, the (111) and (1−11) planes (left and top faces). The adjacent domains have clearly different natures that have to be related to the mechanism of grain formation.

**Figure 3:**
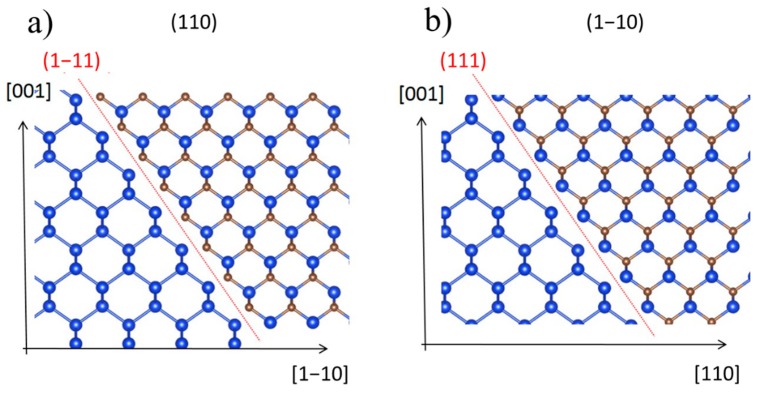
Projections of Si/SiC bi-crystal interface on (110) (**a**) and (1−10) (**b**) planes. The Red line represents the {111} plane and the interface between Si and SiC crystals.

To give more insight into the nature of the domains, we first consider that in the silicon crystal, where pyramids are fabricated, the four {111} planes ((111), (−111), (1−11), (−1−11)) are equivalent while in 3C-SiC crystal they are not. They, indeed, have different polarities. In 3C-SiC monocrystal, (111) and (−1−11) faces expose (for example) silicon atoms and are called “silicon faces” while (1−11) and (−111) expose carbon atoms and are called “carbon faces”. This means that in a Si-SiC bi-crystal, the silicon pyramid in (111) plane should “couple” with carbon face of 3C-SiC while silicon (1−11) face should “couple” with silicon face. These two interfaces are clearly different in nature and are expected to have different properties as, for example, growth rate. To make it clearer, Figure 3 is drawn. In Figure 3a, the SiC/Si bi-crystal projected along the (110) plane is represented. Here, the interface between SiC and Si realizes a Si-C bond perpendicular to the (1−11) plane (red line). In Figure 3b, the same bi-crystal is projected along the (1−10) plane; here, the interface is realized by a Si-Si bound perpendicular to the (111) plane (red line). It is worth noting that the bi-crystal is the same, and the only difference between Figure 3a,b is the projected plane (i.e., SiC is a monocrystal inside the pyramid).

Interestingly, a closer inspection of Figure 2 reveals that the grains in adjacent faces of the pyramid have similar properties: the morphology of the SiC grown on the (111) and (1−11) are very similar although the two faces should have different polarity and growth rate. It indicates that the growth rate along the two adjacent (left and top) faces was equal (within the experimental errors). The surface free energy of the C faces (300 erg/cm^2^) is known to be lower than the free energy of Si faces (2200 erg/cm^2^) [12]. Thus, we should expect that the C-face grows faster than the Si-face, and we should expect an asymmetry in the pyramid morphology if a monocrystal is grown on a pyramid. On the contrary, symmetric pyramids with grain boundaries were experimentally observed. 

This fact corroborated the speculation that the two adjacent faces ((111) and (1−11)) have the same polarity. Now, we need to be reminded that in 3C-SiC grown on “on-axis” flat silicon, Si-C diatom can orient in two different ways. This leads to two different grain orientations: rotated 180° around the [110] direction (flipped upside down). Grain boundaries separating grains having the above-cited property are called anti-phase boundaries (APBs). It is one of the most common extended defects in 3C-SiC grown of Si [17,20,21,22,23]. 

As evidenced in Figure 2, face (111) and face (1−11) belong to different crystals (a grain boundary between these two regions is apparent from SEM image) and have the same polarity (they have the same growth rate). Therefore, APB develops between adjacent pyramid sides.

In this case, at the Si/SiC interface, we should have only one kind of configuration shown in Figure 3. Nevertheless, at this stage, we are not able to distinguish if the interface structure is similar to Figure 3a or Figure 3b. Thus, we performed cross-section STEM images of the interface between SiC/Si on the faces of the pyramid. 

**Figure 4:**
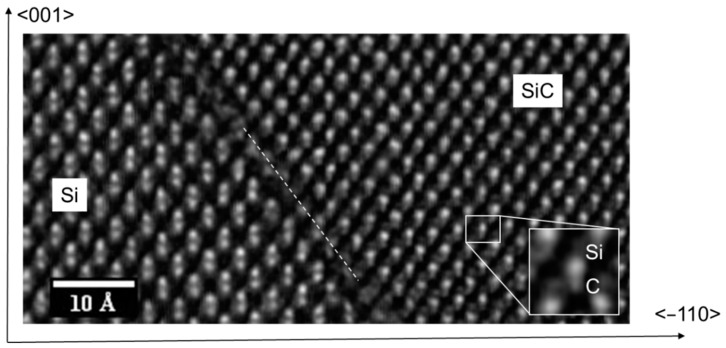
High-resolution STEM image of the heterointerface along the pyramid face. Si/SiC interface is projected on (110) plane. The white dotted line represents the interface between Si and SiC crystals. In the lower right part, a magnification of the silicon and carbon diatom is shown.

High-resolution STEM investigation was performed, and, as an example, an image of the hetero-interface is shown in Figure 4. In this image, the hetero-interface is indicated with a dotted line. On the left, the Si-Si doublet is apparent while on the right typical 3C-SiC ABAC stacking under [110] zone axis is shown, and the Si-C diatom is evident. In the lower right part, a magnification of the Si-C diatom is reported.

This image sheds light on the (111) SiC orientation near the interface and the polarity of the exposed faces on the pyramid. As observed, the more favorable configuration is that shown in Figure 3a. The Si/SiC interface, in both adjacent sides of the pyramid, exposes a carbon face at Si/SiC interface, and thus, we have a Si–C bond perpendicular to the (111) (red line) plane. We can also extrapolate that at the (111) SiC/air interface always expose a “silicon face” when SiC is grown on silicon.

### 3.2. APB Formation and Reduction

The morphology of the SiC grown on ISP is dependent on both the thickness of the epilayer and the size of the pyramid. In Figure S1, we present some SEM images of SiC epilayer grown on Si. Samples have different thicknesses and pyramid size. In particular, Figure S1a,b are obtained growing 1 μm and 10 μm, respectively, on ISP of 5 × 5 µm^2^ in size. In Figure S1b, holes are apparent and appear in correspondence to the vertex of the pyramid. Their nature will be discussed later in the paper. Their presence reduces the quality of the epilayer. To eliminate the holes, we reduced the size of the pyramid. In Figure S1c,d, we show the SEM plan view of 6 μm thick epilayers grown on 3 × 3 µm^2^ and 1 × 1 µm^2^ pyramid respectively. In the 3 × 3 µm^2^ sized pyramid, the holes were still apparent while in the 1 × 1 µm^2^ their presence was avoided. The overall quality of the layer is clearly superior. In the same image, the brighter lines that furrow the surface are the APB, as already reported. 

After eliminating the holes, the quality of the epilayer was limited by the presence of APB. Indeed, APB is known to be a device killer defect in 3C-SiC [17]. Thus, we focused our efforts on the reduction of APBs. To eliminate these boundaries, we changed the growth conditions. In the first trials, we modified the C/Si ratio during the growth step. In Figure S2, we show some examples of SEM plan view of samples grown with the C/Si ratio between 1.2 and 3.15. A sample with less GB density was grown by using a C/Si ratio of 1.6. It is worth noting that for 1.2 and 1.6, the morphology is almost unchanged, but for C/Si = 3.15, a high concentration of GB and small holes were apparent. Raman and XRD results (not showed here) suggest that a high C/Si ratio created carbonic features on the surface that reduce the morphology quality. C/Si ratio of 1.6 was chosen to be the optimized one. 

In a second attempt to reduce the grain boundary density, we changed the growth pressure from 80 to 150 mbar, but no remarkable difference could be observed. 

**Figure 5:**
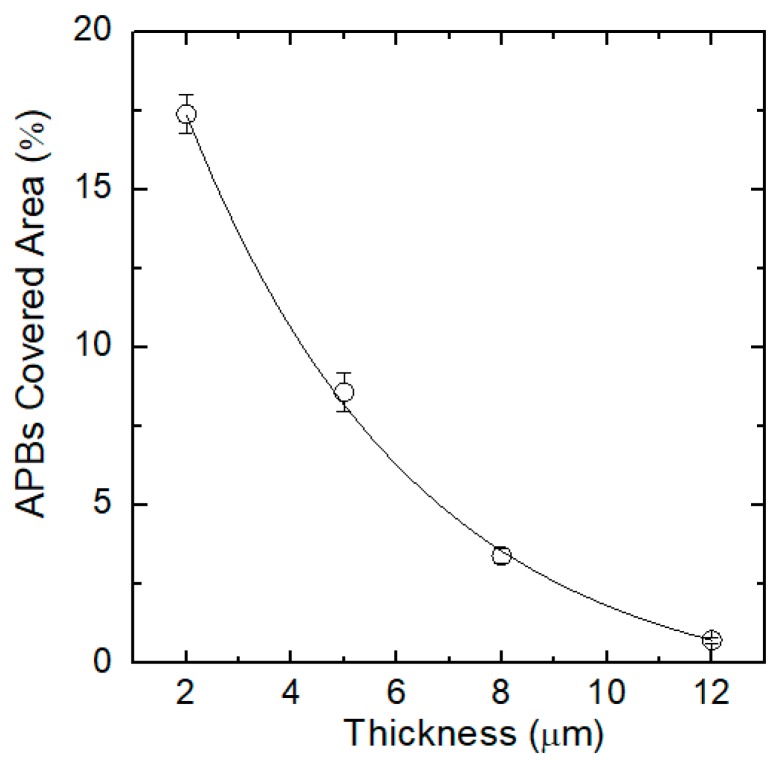
Anti-phase boundaries (APBs) covered area percentages for different thicknesses of epitaxial growth.

Grain boundaries can be highly reduced, increasing the film thickness. In Figure S3, the SEM images of 2 μm thick and 6 μm thick SiC, grown on 1 × 1 µm^2^ pyramid are shown as an example. It is apparent that the 2 μm thick layer had a higher value of grain boundary length (for unit surface) with respect to the 6 μm thick layer. From plan view SEM analysis, we estimated the APBs covered area as a function of the layer thickness (Figure 5). Although the ISP compliant substrate generates APBs (from the coalescence of 3C-SiC, coming from orthogonal {111} faces), a rapid exponential decay in APBs concentration was achieved after a few microns of growth. In particular, it can be noted that in a 12 μm epitaxy, the APBs coverage was reduced by a factor 20 with respect to the initial layers. This suggests that increasing the layer thickness is an efficient way to lower the APBs concentration leading to a uniformly smooth surface sample morphology. 

### 3.3. GB Interaction and Void Formation

**Figure 6:**
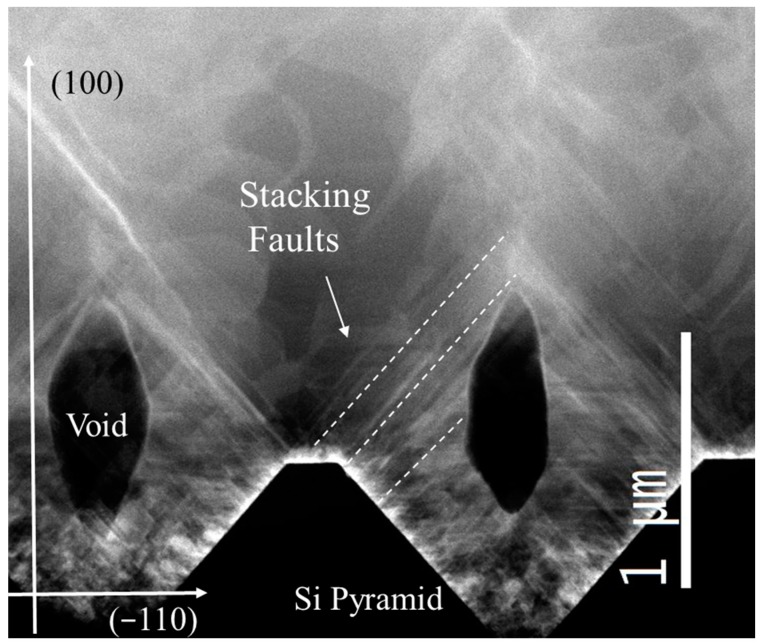
TEM plan view of a 12 μm-thick sample. Stacking faults are indicated together with major crystallographic directions. Si pyramid, voids, and SFs are indicated.

Although the quality of the epilayer can be greatly increased by increasing the thickness, the ISP morphology induced the formation of buried voids in the epilayer. These voids are observed in STEM cross images in a high thickness epilayer and are related to the holes observed in the low thickness epilayer of Figure S1. In Figure 6, we show a cross-section STEM image of 12 µm-thick sample growth on ISP 1 × 1 μm^2^. Si pyramids are shown in the bottom of the image in black, while SiC is related to the brighter region. Several straight lines inclined at about 57° are SFs, while curved bright lines are grain boundaries. On top of the vertex of each silicon pyramid, the presence of a void is apparent. On the one hand, the role of the void can be beneficial for the epilayer quality, and it could, in fact, relax the stress related to the Si/SiC hetero-interface and more importantly, can annihilate the SFs nucleating on the Si pyramid side. Indeed, the SFs that nucleated in the {111} planes of the Si pyramid collided with the void and were eliminated. Nevertheless, SFs that nucleated in the top part of the pyramid and in the (001) zone were able to surmount the void and propagate in the sample. As an example, some of these SFs are highlighted in Figure 6. Unfortunately, these SFs can reach the surface. 

**Figure 7:**
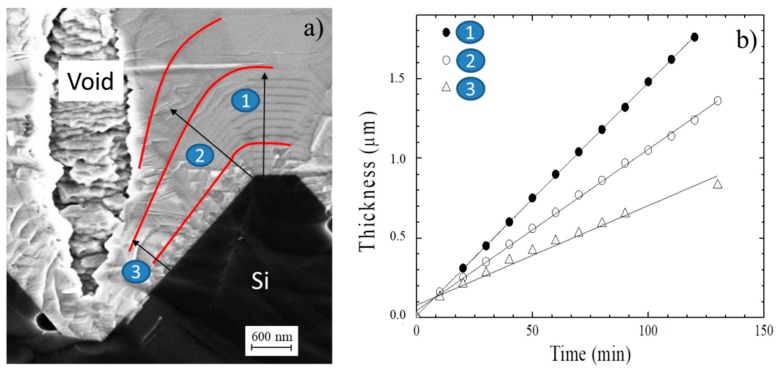
(**a**) SEM cross-section of the pyramid showing the void and the N^+^ markers. Red lines are drawn as a guide for the eyes. In the same image three black straight lines are drawn: Line 1 is related to the growth of (001) SiC plane, while lines 2 and 3 refer to the growth of (111) plane in two different positions of the pyramid; (**b**) The thickness of the epilayer as a function of growth time for line 1, 2, and 3 of the figure (**a**).

To give more insight into the structure of the void and into the motivation of this formation, a series of new experiments was performed. In such experiments, N_2_ gas was introduced into the reaction chamber every 10 min during growth. This creates highly doped markers and allows the reconstruction of the surface front during growth. In Figure 7a, an SEM cross-section of the pyramid showing the void, and the N^+^ markers are shown. Red lines follow the N^+^ profile, and they are drawn as a guide for the eyes. In the same image, three black straight lines are drawn: Line 1 is related to the growth of (001) SiC plane, while lines 2 and 3 refer to the growth on (111) plane in two different positions of the pyramid.

It is worth noting that it is possible to measure the growth rate along with the 1, 2, and 3 lines. In Figure 7b, we show the thickness of the film as a function of growth time along these lines. The growth rates were 860 ± 4, 600 ± 4, and 370 ± 4 nm/h for 1, 2, and 3 lines, respectively. The growth rate of the (001) was about 40% higher than the growth rate of the (111), as expected. The extremely interesting fact is that the growth rate depends on the position on the (111) plane if it is measured on line 2 or 3. From easy geometrical considerations, a conformal growth could be observed if the ratio between (111) and (001) growth rates is about 0.81. A scheme is represented in Figure S4. Higher values of this parameter lead to the filling of the pyramid while lower values, to the formation of voids. In our case, the ratio was 0.69 (<0.81) for line 2, so the formation of voids was geometrically allowed. Moreover, the growth rate in the (111) plane depended on the distance from the vertex; indeed, the nearer the pyramid vertex, the lower the grown rate. (0.69 and 0.43 for line 2 and 3, respectively). This fact could be explained considering that deeper structures suffer from lower gas diffusion, and a reduction in the growth rate is possible. It is also worth noting that at the pyramid edges, APBs develop, and four APBs meet in the vertex of the pyramid (as shown in Figure 2b). It is going to be demonstrated in a separate paper that the growth rate near an APB is highly reduced, creating a furrow on the surface. This should happen even near the vertex of the pyramid, where four GBs meet. Here, a large reduction in the growth rate is probable. This, thus, increasingly reduces the (111)/(001) growth rate ratio forming the void. 

**Figure 8:**
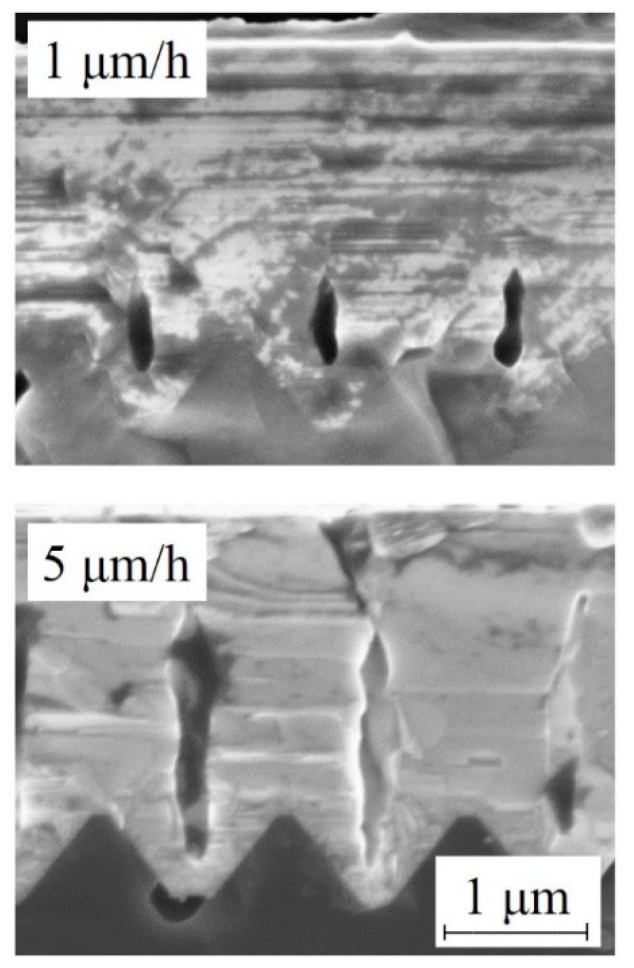
Cross SEM images of the SiC layer grown on 1 × 1 µm^2^ ISP obtained with different growth rates. Scale bar is the same for both the images.

As already reported, the formation of the void can be beneficial for the reduction of the defectiveness of the epilayer. Voids, in fact, can annihilate SFs. Control of the void height and width is needed to reduce the SFs. In a new series of experiments, the growth rate was changed, acting on the gasses fluxes. In Figure 8, we show cross SEM images of the SiC layer grown on 1 × 1 µm^2^ ISP obtained with different growth rates. This figure demonstrates that we are able to control the void height changing the growth rate. As an example, films grown at 1 and 5 μm/h had a void height of 0.6 μm and 1.6 μm, respectively. In the last case, all the SFs “starting” from the Si/SiC heterointerface were eliminated. 

## 4. Conclusions

In the present paper, we studied the effect of a compliant substrate able to reduce the defectiveness of 3C-SiC grown on Si. In particular, we patterned a Si wafer with inverted silicon pyramids of different sizes and pitch. We investigated the effect of the sizes on the epilayer quality. We noted that anti-phase boundaries developed between pyramid adjacent faces and that SiC/Si interface had the same polarity on both pyramid faces. This induced SiC growth on ISP to always have a silicon face exposed to air. Moreover, we observed the presence of voids buried in the epilayer that form due to the merging of APB in the vertex of the pyramid. We studied how to control the height of the void and the density of APB. Finally, we exploited APBs and SFs elimination mechanisms to improve the quality of the epilayer.

## Figures and Tables

**Table 1:** Size and Pitch of the Manufactured Pyramids.

Pyramid Size	Pitch
5 × 5 μm^2^	6 × 6 μm^2^
3 × 3 μm^2^	4 × 4 μm^2^
1 × 1 μm^2^	1.4 × 1.4 μm^2^

## Supplementary Materials

The following are available online at https://www.mdpi.com/1996-1944/12/20/3407/s1, Figure S1: Plan view SEM image of SiC layer grown on ISP. (**a**) Pyramid size 5 × 5 μm^2^ and layer thickness 1 μm; (**b**) Pyramid size 5 × 5 μm^2^ and layer thickness 10 μm; (**c**) Pyramid size 3 × 3 μm^2^ and layer thickness 6 μm; (**d**) Pyramid size 1 × 1 μm^2^ and layer thickness 6 μm. Holes and grain boundaries (GB) are indicated; Figure S2: SEM plan view images of samples grown with a C/Si ratio between 1.2 and 3.15. Pyramid size was 1 × 1 μm^2^; Figure S3: SEM plan view images of 2 μm and 6 μm thick 3C-SiC grown on 1 × 1 μm^2^ pyramid; Figure S4: Ratio between (111) and (001) growth rates generating 3 different types of epilayer morphologies.
