# Supplementary material for: 3C-SiC Growth on Inverted Silicon Pyramids Patterned Substrate
Source: Materials (Basel). 2019 Oct 18;12(20):3407. doi: 10.3390/ma12203407 (PMC6829442; doi:10.3390/ma12203407)
Supplement: Supplementary file 1 [file materials-12-03407-s001.pdf]

materials

MDPI

Article

3C-SiC Growth on Inverted Silicon Pyramids Patterned Substrate

Massimo Zimbone [1], Marcin Zielinski [2], Corrado Bongiorno [3], Cristiano Calabretta [3,*], Ruggero Anzalone [4], Silvia Scalese [3], Giuseppe Fisicaro [3], Antonino La Magna [3], Fulvio Mancarella [5] and Francesco La Via [3]

1 CNR-IMM, V. S.Sofia 64, 95129 Catania, Italy; massimo.zimbone@imm.cnr.it
2 Savoie Technolac-Arche Bat.4, Allée du Lac d'Aiguebelette, BP 267, 73375 Le Bourget du Lac Cedex, France; mzielinski@novasic.com
3 CNR-IMM, Zona Industriale VIII Strada 5, 95121 Catania, Italy; Corrado.Bongiorno@imm.cnr.it (C.B.); Silvia.Scalese@imm.cnr.it (S.S.); giuseppe.fisicaro@imm.cnr.it (G.F.); antonino.lamagna@imm.cnr.it (A.L.M.); francesco.lavia@imm.cnr.it (F.L.V.)
4 STMicroelectronics, Stradale Primosole, 50, 95121 Catania, Italy; ruggero.anzalone@st.com
5 CNR-IMM, Via Gobetti 101, I-40129 Bologna, Italy; mancarella@bo.imm.cnr.it
* Correspondence: cristiano.calabretta@imm.cnr.it

Received: 21 September 2019; Accepted: 16 October 2019; Published: 18 October 2019

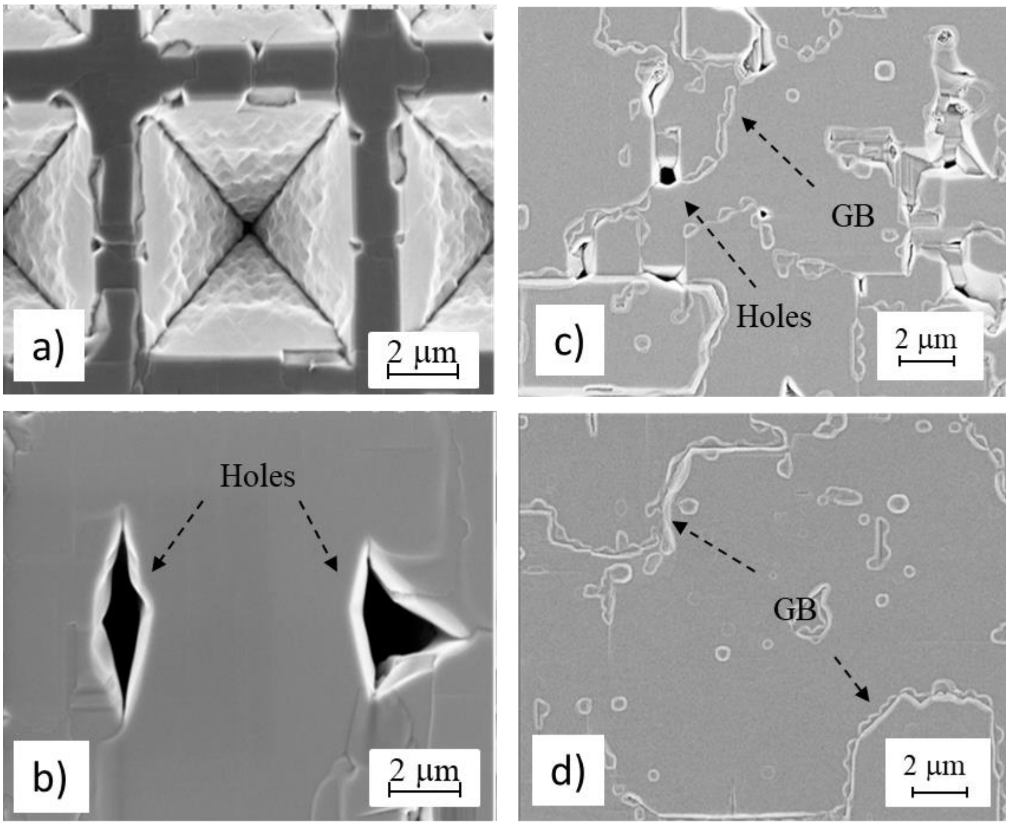

Figure S1. Plan view SEM image of SiC layer grown on ISP. (**a**) Pyramid size 5 × 5 μm² and layer thickness 1 μm; (**b**) Pyramid size 5 × 5 μm² and layer thickness 10 μm; (**c**) Pyramid size 3 × 3 μm² and layer thickness 6 μm; (**d**) Pyramid size 1 × 1 μm² and layer thickness 6 μm. Holes and grain boundaries (GB) are indicated.

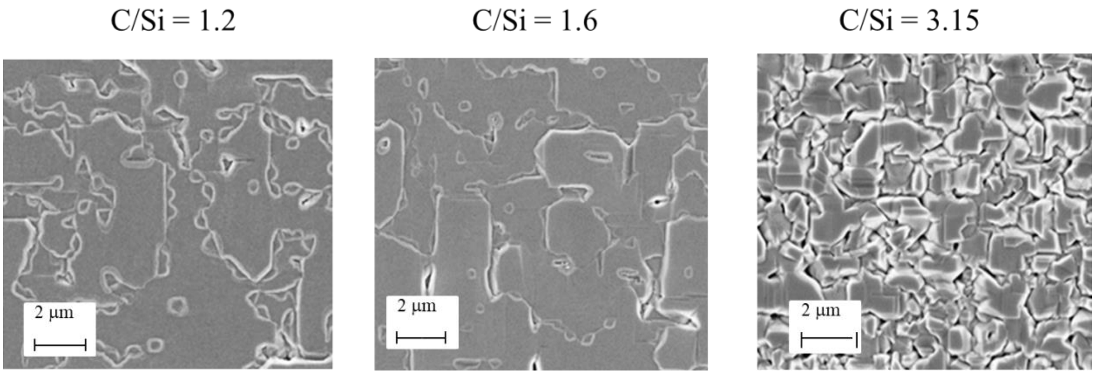

Figure S2. SEM plan view images of samples grown with a C/Si ratio between 1.2 and 3.15. Pyramid size was 1 × 1 µm^2.

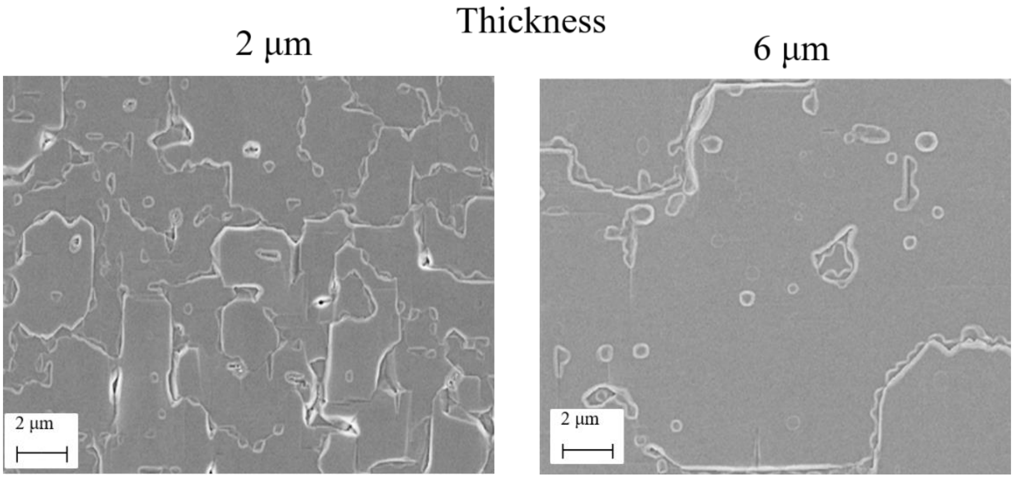

Figure S3. SEM plan view images of 2 µm and 6 µm thick 3C-SiC grown on 1 × 1 µm^2 pyramid.

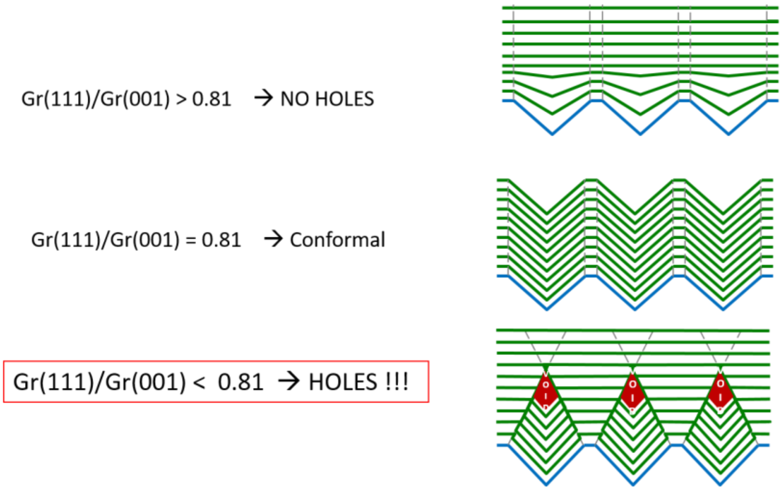

Figure S4. Ratio between (111) and (001) growth rates generating 3 different types of epilayer morphologies.
